# Protection from experimental cerebral malaria with a single intravenous or subcutaneous whole-parasite immunization

**DOI:** 10.1038/s41598-018-21551-2

**Published:** 2018-02-15

**Authors:** Kirsten Heiss, Marion Irmgard Maier, Angelika Hoffmann, Roland Frank, Martin Bendszus, Ann-Kristin Mueller, Johannes Pfeil

**Affiliations:** 1 Centre for Infectious Diseases, Parasitology Unit, Heidelberg University Hospital, Heidelberg, Germany; 2 Centre for Childhood and Adolescent Medicine, General Pediatrics, Heidelberg University Hospital, Heidelberg, Germany; 3 German Centre for Infection Research (DZIF), Heidelberg, Germany; 4 Department of Neuroradiology, Heidelberg University Hospital, Heidelberg, Germany; 5 Division of Experimental Radiology, Department of Neuroradiology, Heidelberg University Hospital, Heidelberg, Germany; 6 MalVa GmbH, Heidelberg, Germany

## Abstract

Cerebral malaria is a life-threatening complication of *Plasmodia* infection and a major cause of child mortality in Sub-Saharan Africa. We report that protection from experimental cerebral malaria in the rodent model is obtained by a single intravenous or subcutaneous whole-parasite immunization. Whole-parasite immunization with radiation-attenuated sporozoites was equally protective as immunization with non-attenuated sporozoites under chemoprophylaxis. Both immunization regimens delayed the development of blood-stage parasites, but differences in cellular and humoral immune mechanisms were observed. Single-dose whole-parasite vaccination might serve as a relatively simple and feasible immunization approach to prevent life-threatening cerebral malaria.

## Introduction

Malaria remains a major cause of global morbidity and mortality. By current estimates, severe malaria kills roughly 1200 persons per day^1^. Most of these fatalities occur due to cerebral malaria (CM)^2^, which is the most severe complication associated with *Plasmodium falciparum* (*Pf*) infection. CM is characterized by altered consciousness and coma^3^, and disproportionally affects young children in sub-Saharan Africa. At older ages, after as few as one or two malaria infections, people living in endemic areas develop a partial immunity against blood stages of malaria infection and thereby become protected against severe complications^4^.

In recent years, the intensity of malaria transmission in Africa has declined due to the widespread use of insecticide-treated bed nets and other vector control measures. At the same time, declining transmission intensity delays the acquisition of immunity, and there is evidence that CM becomes a more common presentation in children living in lower transmission areas^5^. Consequently, there remains an urgent need to develop novel therapies to prevent CM.

Immunization by administration of attenuated whole-parasites is currently under clinical investigation to induce complete, sterile protection against malaria infection. Complete protection is mediated at the pre-erythrocytic stages of parasite development, and is particularly dependent on high numbers of cytotoxic T-cells eliminating *Plasmodia* during liver stage development (reviewed in^6^). Moreover, recent data suggests that immunization with irradiated sporozoites (radiation-attenuated sporozoites, RAS) may not only result in immune responses against pre-erythrocytic, but also against blood-stage antigens^7,8^. Blood-stage directed immune responses were also reported following repeated immunization with sporozoites under chloroquine prophylaxis (chloroquine-chemoprophylaxis with sporozoites; CQ-CPS) in rodent models^9,10^ and in human vaccination trials^11,12^.Although blood-stage directed immunity was considered negligible in vaccination trials that intended to achieve complete protection from malaria infection^12,13^, blood-stage directed immune responses could mitigate the clinical course of malaria infection. In the case of infection, this would mean that whole parasite immunization would still protect vaccinated individuals against the development of severe, life-threatening malaria.

Here, we demonstrate that a single RAS or CQ-CPS immunization induces functional blood-stage directed immunity, which mitigates the clinical course of malaria and prevents fatal experimental cerebral malaria (ECM) in the rodent model.

## Results

### A single intravenous or subcutaneous whole-parasite immunization protects mice against ECM

**Figure 1:**
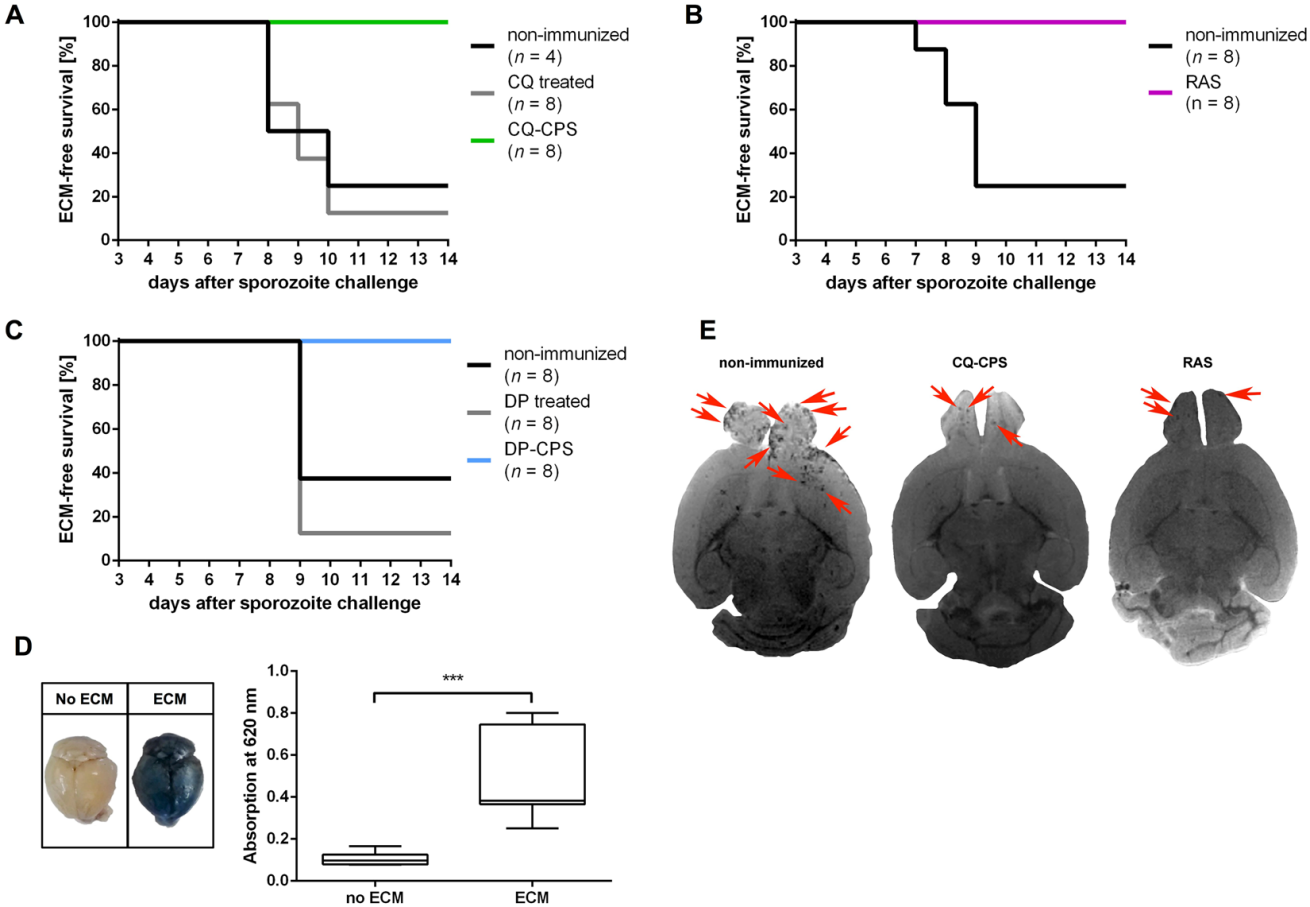
A single CPS or RAS immunization protects mice against experimental cerebral malaria. (**A**) ECM-free survival following a challenge with 10^3^ *Pb* ANKA SPZ i.v. in previously CQ-CPS immunized (n = 8) versus CQ-treated (n = 8) or untreated (non-immunized; n = 4) control animals. (Non-immunized vs. CQ-CPS: P < 0.01; CQ treated vs. CQ-CPS: P < 0.001; Non-immunized vs. CQ treated: P = 0.7, logrank-test). (**B**) ECM-free survival in i.v. RAS immunized (n = 8) versus untreated (non-immunized; n = 8) control animals following a challenge with 10^3^ *Pb* ANKA SPZ i.v. (P < 0.001, logrank-test). (**C**) ECM-free survival in s.c. DP-CPS immunized (n = 8) versus DP treated (n = 8) or untreated (non-immunized; n = 8) animals (Non-immunized vs. DP-CPS: *P* < 0.01; DP treated vs. DP-CPS: *P* < 0.001; Non-immunized vs. DP treated: *P* = 0.26, logrank-test). (**D**) Evans Blue staining of the brains of animals with (right) and without (left) clinical signs of ECM (shown are representative pictures). Following i.v. injection of Evans Blue, significantly higher amounts of Evans Blue were detected in the brains of ECM (n = 10) versus non-ECM (n = 13) animals, (P < 0.001, Wilcoxon rank-sum test). (**E**) Representative MR brain images (MRI assessment) show a high number of microhemorrhages in non-immunized mice with a predominance in the olfactory bulb, while in CQ-CPS and RAS immunized mice only a low number of microhemorrhages is visible (red arrows point to exemplary microhemorrhages).

In order to investigate whether protection against ECM can be achieved after single-dose whole-parasite vaccination, we immunized groups of eight C57BL/6 J mice by a single intravenous (i.v.) administration of CQ-CPS (3 × 10^4^ sporozoites (SPZ), *Plasmodium berghei* ANKA was used in all experiments) or by a single i.v. administration of 3 × 10^4^ RAS. Six weeks after the immunization process, experimental animals were infected i.v. with 10^3^ infectious SPZ. All animals developed blood-stage infection. However, immunized mice survived without developing clinical symptoms of ECM, whereas 60–90% of non-immunized control animals developed ECM at 7 to 9 days after challenge (Fig. 1A and B).

We subsequently questioned if protection against ECM could be obtained not only by i.v., but also by a single subcutaneous (s.c.) SPZ vaccination. Following our previously established concept of single-dose piperaquine chemoprophylaxis (DP-CPS)^14^ and histamine supplementation^15^, mice were immunized s.c. with a single administration of 10^5^ SPZ in conjunction with 100 µg histamine. Due to the extended elimination half-life of piperaquine^16^ animals were challenged with 10^3^ SPZ i.v. 12 weeks after the immunization process. Again, all immunized animals were protected against the onset of ECM whereas 7 out of 8 drug control (DP treated) and 5 out of 8 untreated (non-immunized) animals succumbed to ECM at day 9 after challenge infection (Fig. 1C).

### Evans blue staining and magnetic resonance imaging confirm the absence of severe brain pathology in immunized mice

To complement the clinical assessment, we used Evans Blue staining to confirm the integrity of the blood-brain barrier (BBB) and *ex vivo* magnetic resonance imaging (MRI) to assess microhemorrhages in immunized and ECM-protected mice. Evans Blue was injected i.v. in a subset of 10 non-immunized mice with clinical symptoms of ECM (Rapid Murine Coma and Behavioral Scale < 5)^17^ and 13 RAS or CQ-CPS immunized mice that survived without clinical symptoms of ECM by day 14 after challenge infection. Significant extravasation of the dye to the brain parenchyma was seen in mice with clinical symptoms of ECM, and thus confirmed the presence of BBB disruption in ECM affected mice in contrast to immunized mice that remained protected from ECM and showed no evident Evans Blue extravasation (Fig. 1D). In non-immunized mice with clinical symptoms of ECM, MRI assessment confirmed a high microhemorrhage load in the olfactory bulb (OB), which represents a predilection site of disease in ECM^18,19^. Only few microhemorrhages were present in the OB of CQ-CPS and RAS immunized mice (Fig. 1E).

### Development of blood-stage parasites is impaired after a single RAS or CQ-CPS immunization

**Figure 2:**
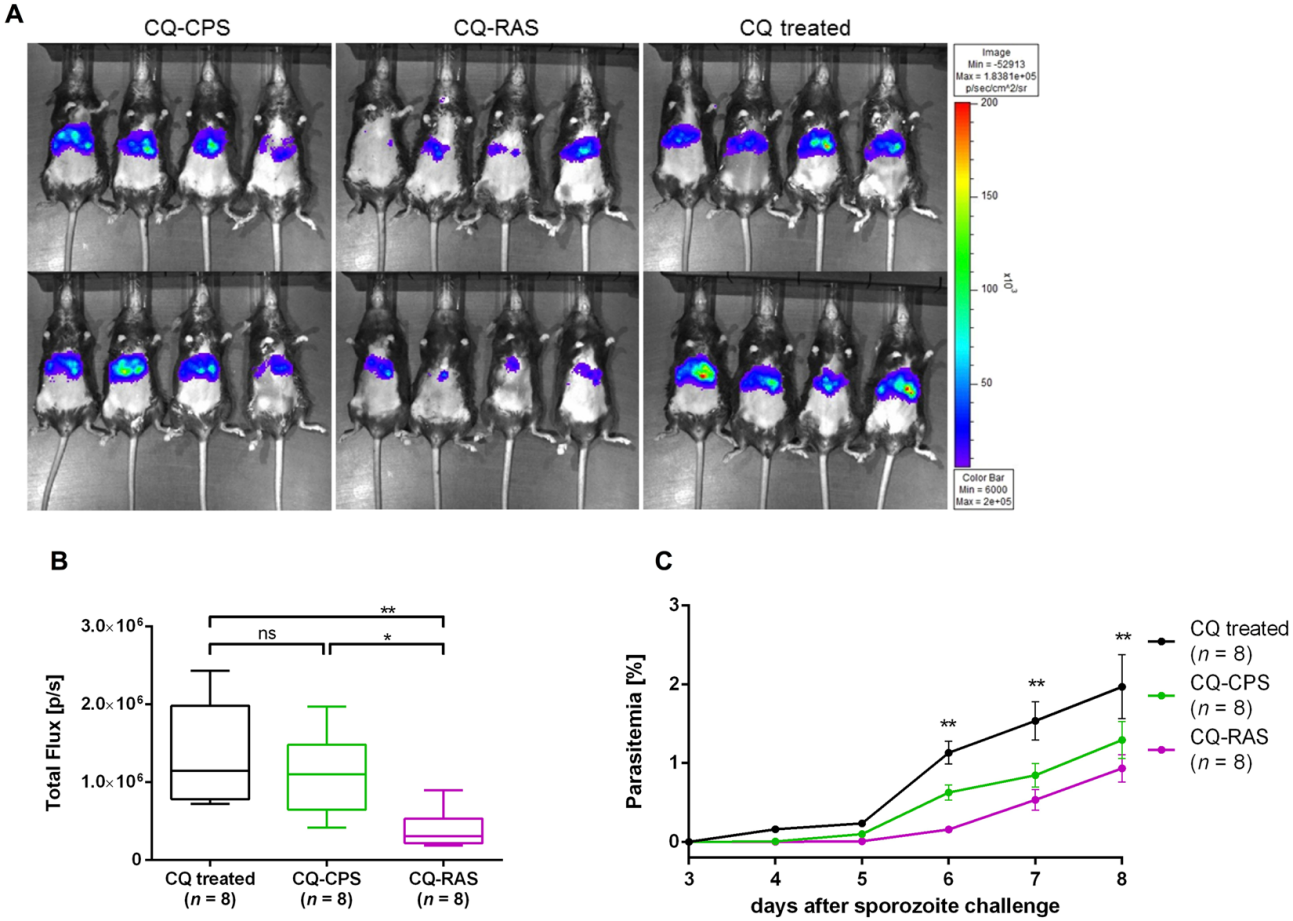
Liver- and blood-stage parasite development after a single RAS or CPS immunization. (**A**) Visualization of *in vivo* parasite liver load 42 h after i.v. challenge with 10^3^ luciferase-expressing *Pb* ANKA SPZ in previously CQ-CPS (n = 8) or CQ-RAS (n = 8) immunized mice versus CQ treated (n = 8) control animals. (**B**) A single CQ-RAS but not CQ-CPS immunization resulted in reduced parasite liver load in comparison to CQ-treated control mice at 42 h after i.v. challenge with 10^3^ luciferase-expressing *Pb* ANKA SPZ (*P < 0.05; **P < 0.01, Tukey’s multiple comparisons test, 1-way ANOVA). (**C**) CQ-RAS (n = 7, one mouse who did not develop parasitemia was excluded from the analysis) and CQ-CPS (n = 8) immunization resulted in significantly impaired blood-stage development on days 6 to 8 post challenge with 10^3^ *Pb* ANKA SPZ i.v. in comparison to CQ-treated (n = 8) control mice (**P < 0.01, repeated measures ANOVA. Within the model, parasitemia on days 6, 7 and 8 after challenge were set as depended variables and analyzed with the covariate of d5 parasitemia).

Attenuated blood-stage vaccines produced both by radiation^20^ or gene disruption^21^ induce protection against ECM in rodent *Plasmodium* infections, which suggests that blood-stage directed immune responses can prevent the pathology of ECM. We therefore questioned if the protection from ECM after single-dose RAS and CPS immunization could be explained by blood-stage directed immunity. We immunized mice under CQ prophylaxis with either 3 × 10^4^ RAS (CQ-RAS) or 3 × 10^4^ SPZ (CQ-CPS) in comparison to mice treated only with CQ (CQ treated). CQ was given to all mice in order to adjust potential CQ effects on blood-stage development and immune responses across experimental groups. Following an infectious challenge with 10^3^ SPZ i.v. six weeks after the immunization, we compared pre-erythrocytic and blood-stage parasite development in immunized versus drug-treated animals. A single immunization with CQ-RAS, but not with CQ-CPS, resulted in a significant decrease in parasite liver load (Fig. 2A and B). Blood-stage parasites became visible 4 days after challenge infection in all CQ-treated control mice. Prepatency was prolonged by 1–2 days in CQ-RAS immunization mice. Furthermore, immunization with both CQ-CPS and CQ-RAS affected blood-stage growth, which led to significantly lower parasitemia levels on days 6 to 8 after challenge (Fig. 2C).

### Stage-transcending protection from ECM after a single immunization with RAS

**Figure 3:**
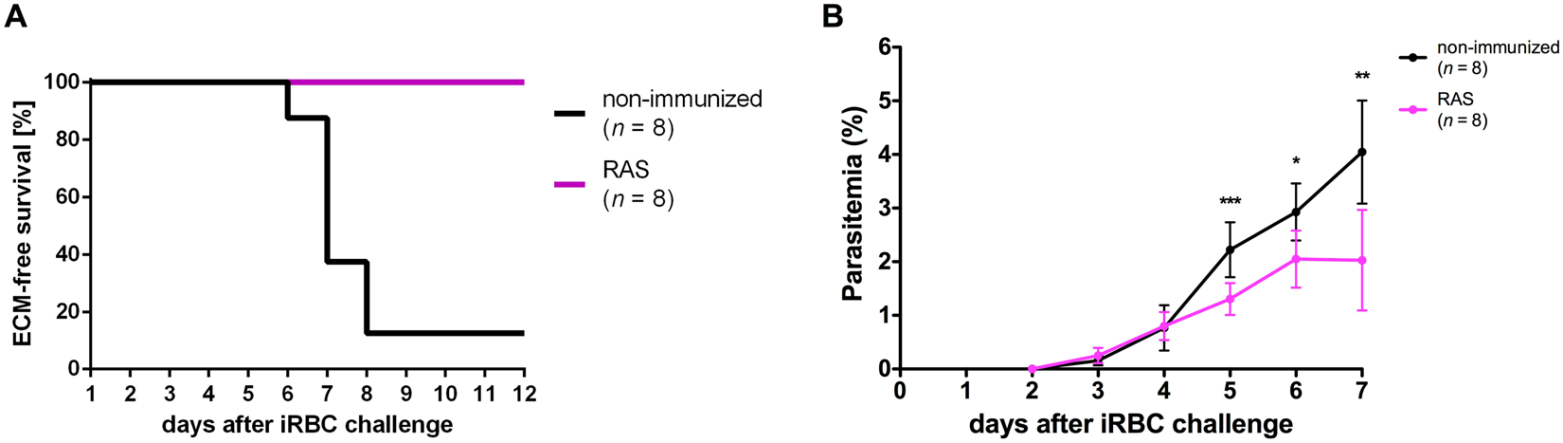
Protection from ECM and parasitemia in RAS immunized mice after challenge with infected red blood cells (iRBC). (**A**) A single i.v. RAS immunization (n = 8) protected animals from ECM after challenge with 10^5^ iRBC (***P < 0.001, logrank test). (**B**) Following a challenge with 10^5^ infected red blood cells, the development of blood stage parasites parasitemia was decelerated in RAS immunized mice resulting in significantly lower parasitemia on days 5 to 7 after challenge in comparison to mock-control mice (*P < 0.05, (**P < 0.01, ***P < 0.001, Mann-Whitney-U-test).

In order to test the hypothesis that a single administration of RAS induced a stage-transcending, blood-stage directed immune response, we immunized another group of mice with 3 × 10^4^ RAS. Mock control mice (CQ-Mock group) were given equal amounts of CQ, and were injected with equivalent amounts of uninfected salivary gland tissue. Six weeks after the immunization, mice were challenged with 10^5^ *Pb* ANKA infected red blood cells (iRBC), thereby bypassing pre-erythrocytic parasite development. In this experiment, all RAS-immunized mice remained protected from ECM, whereas 7 out of 8 non-immunized (Mock-control) mice developed ECM (Fig. 3A). Significantly lower parasitemia was observed in RAS-immunized mice starting from day 5 after iRBC challenge (Fig. 3B).

We corroborated this finding in a second experiment where groups of eight mice were immunized by administering CQ-RAS or CQ-CPS. Six weeks after the immunization, mice were infected with 10^5^ *Pb* ANKA infected red blood cells (iRBC). In good agreement with the previous results, we found that 88% (7/8) CQ-RAS or CQ-CPS immunized mice survived without developing clinical symptoms of ECM, compared to 25% (2/8) surviving CQ-Mock control mice (Fig. S2). Parasitemia increased at a lower rate in CQ-RAS and CQ-CPS immunized mice, resulting in significantly lower parasitemia levels on days 4 and 5 after infection with 10^5^ iRBCs in comparison to the CQ-Mock control animals. We could not observe any significant difference in parasitemia between CQ-RAS and CQ-CPS immunized mice (Fig. S3).

**Figure 4:**
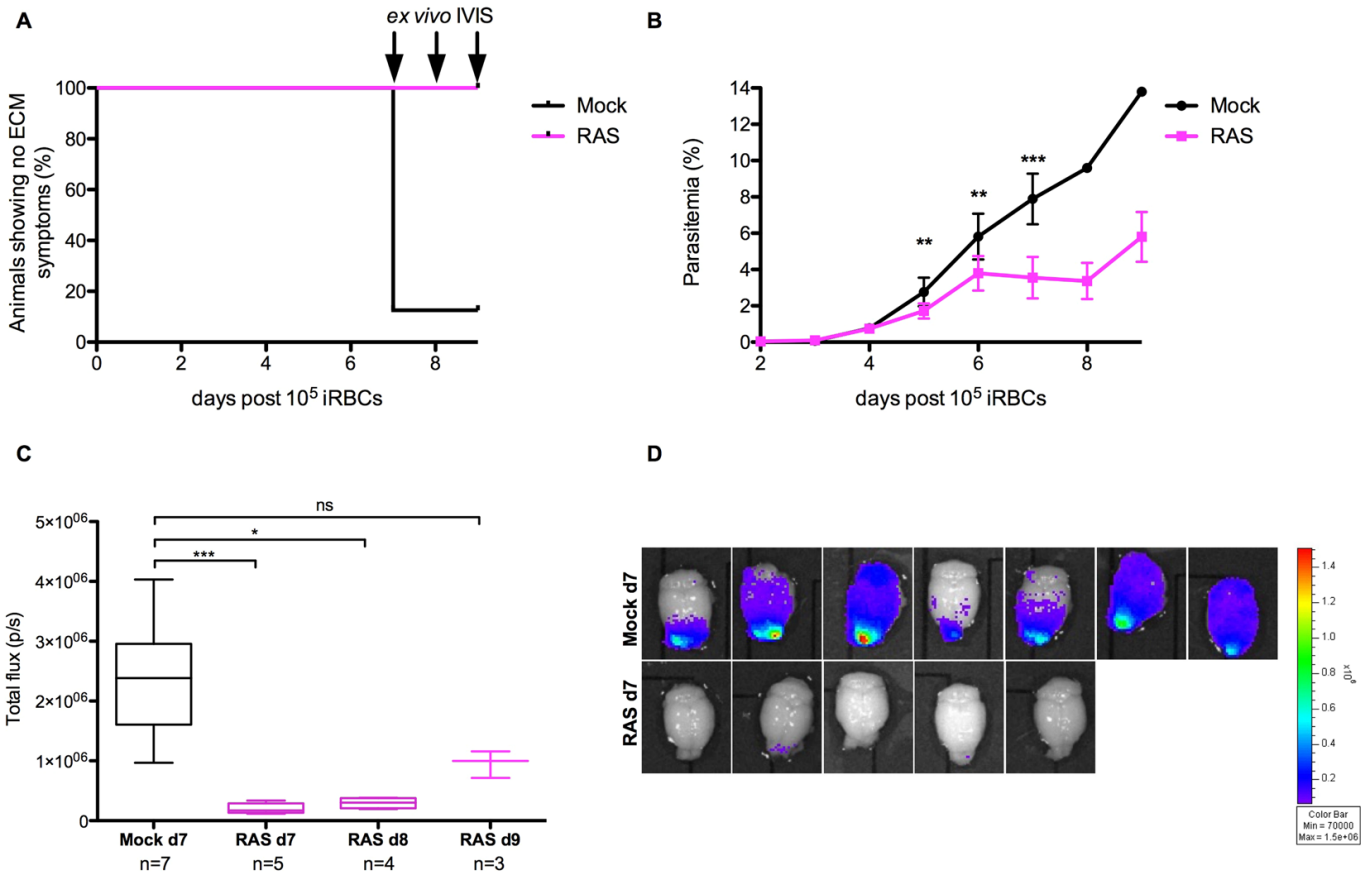
Parasite sequestration in the brain differs between Mock-control and one-shot RAS-immunized mice after iRBC challenge. C57BL/6 J mice were immunized with one-shot of 3 × 10^4^ RAS (n = 12) or salivary gland debris (Mock; n = 8) and challenged 6 weeks later with 10^5^ iRBC of *Pb*GFP Luc_con_ WT parasites. (**A**) One-shot RAS-immunized and iRBC-challenged animals do not develop ECM. (**B**) Delayed blood-stage growth in one-shot RAS-immunized animals compared to Mock-control mice after a challenge with 10^5^ iRBCs. For statistical analysis, a Mann-Whitney Test was applied; day 5, P = 0.002; day 6, P = 0.002 and day 7, P = 0.0002). (**C**) From day 7 to day 9 after infection, one-shot RAS-immunized mice showed a significantly reduced brain parasite load after a challenge with 10^5^ iRBCs. Groups of mice were sacrificed at the day when Mock-injected mice showed ECM (day 7 after challenge; n = 7 for Mock-injected mice and n = 5 for one-shot RAS-vaccinated mice). For RAS-vaccinated animals, additional groups of mice were sacrificed at day 8 (n = 4) and day 9 (n = 3) after challenge. Bioluminescence was recorded as total flux (photons (p)/second (s)). Statistical analysis was performed using Kruskal-Wallis with Dunn’s Multiple Comparison Test (***p < 0.001; **p < 0.01; ns, not significant). (**D**) *Ex vivo* bioluminescence images from RAS and Mock animals at day 7 after challenge. Note the increased bioluminescence signal in ECM-affected Mock animals, which is notable particularly in in the olfactory bulb.

In an additional immunization experiment, we determined the brain parasite load in RAS immunized (and ECM-protected) versus Mock (ECM positive) control mice after a challenge with iRBCs. From days 7 to 9 after infection, one-shot RAS immunized mice consistently showed a significantly reduced sequestration of parasites in the brain as shown by *ex vivo* bioluminescence quantification of iRBC accumulation (Fig. 4). In good agreement, a recent study reported reduced parasite sequestration in the brain of ECM-resistant Interferon regulatory factor 1 deficient mice^22^.

### Humoral and cellular immune responses in CQ-CPS and CQ-RAS immunized mice

**Figure 5:**
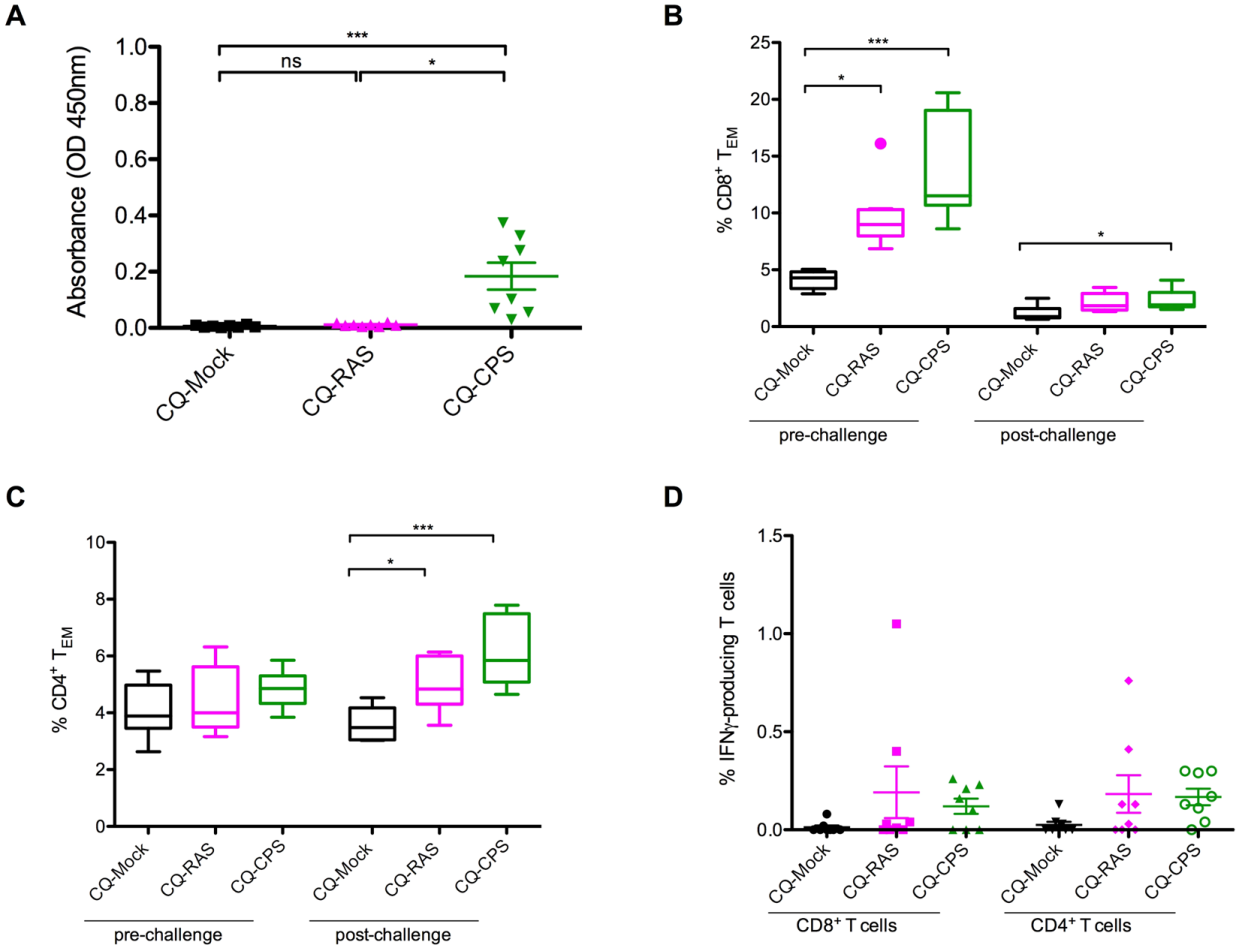
Humoral and cellular immune responses in CQ-RAS and CQ-CPS immunized mice versus CQ-Mock controls. (**A**) Antibody responses to late blood-stage lysates in CQ-RAS (n = 8), CQ-CPS (n = 8) and control mice (n = 8) 14 days before challenge. Sera of immunized and control mice were analyzed by ELISA. (**B**) and (**C**) Flow cytometric assessment of the CD8^+^ T cell (B) or CD4^+^ T cell (**C**) activation status in CQ-RAS (n = 8) and CQ-SPZ (n = 8) immunized mice versus CQ-Mock controls (n = 8). Samples were collected 2 weeks before (pre-challenge) or 4 days after (post-challenge) challenge with 10^5^ iRBC. (**D**) Flow cytometric assessment of IFNy-producing T cells after stimulation with blood-stage lysates in CQ-RAS (n = 8) and CQ-SPZ (n = 8) immunized mice versus CQ-Mock controls (n = 8) 2 weeks before challenge (pre-challenge). IFNy responses from non-stimulated cells (background) were subtracted from stimulated values. *P < 0.05; **P < 0.01; ***P < 0.001 (Kruskal- Wallis test with Dunn’s Multiple Comparison Test).

We measured IgG antibody levels against malaria blood-stage lysates in sera obtained from CQ-RAS, CQ-CPS and CQ-Mock immunized mice before iRBC challenge. Blood-stage specific antibodies were detected in CQ-CPS but not in CQ-RAS immunized mice (Fig. 5A).

We further assessed cellular immune responses by detection of CD44^high^CD62^low^ T effector memory cells (T_EM_) before and after challenge with 10^5^ iRBCs in the peripheral blood. Before challenge, we observed an increased frequency of effector memory CD8^+^ T-cells both in CQ-RAS and CQ-CPS immunized mice compared to CQ-Mock-immunized animals (Fig. 5B). No differences were detected in the CD4^+^ T effector memory cell population (Fig. 5C). On day 4 after challenge with iRBCs, the percentage of effector memory CD8^+^ T-cells decreased in all groups, but remained at significantly higher levels in CQ-CPS mice in comparison to the CQ-Mock control (Fig. 5B). Within the CD4^+^ T-cell population, the frequency of effector memory T-cells increased during the ongoing infection both in CQ-RAS or CQ-CPS immunized animals (Fig. 5C).

We also analyzed the rate of IFNγ-producing peripheral CD8^+^ and CD4^+^ T cells before challenge. After stimulation with blood stage lysate, we detected an increased frequency of cytokine-producing CD8^+^ T and CD4^+^ T cells in CQ-RAS and CQ-CPS immunized mice (Fig. 5D). We noted a wide inter-individual variability, and therefore no statistical significance was obtained in this assessment.

**Figure 6:**
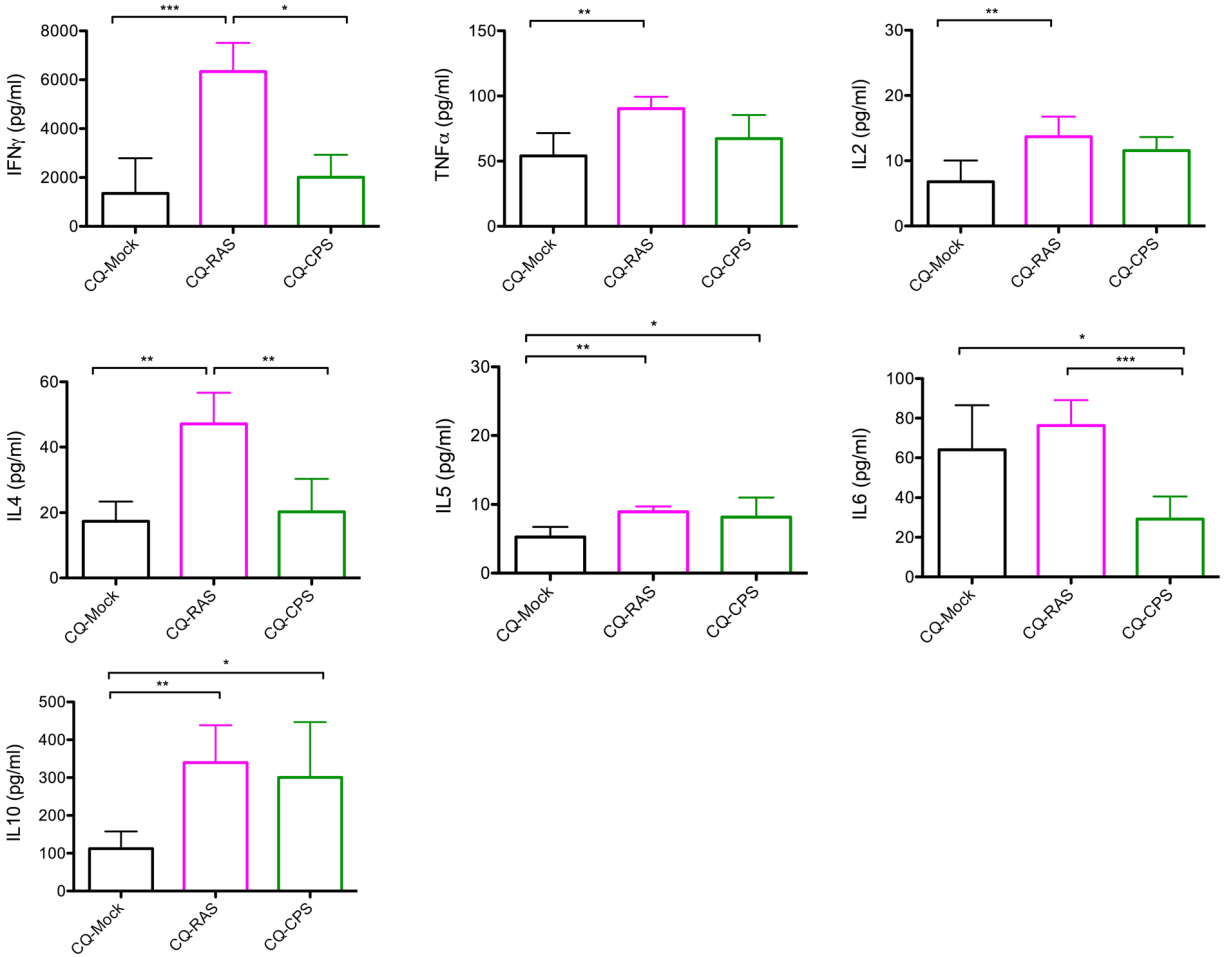
Assessment of cytokine levels in CQ-RAS (n = 8) and CQ-SPZ (n = 8) immunized mice versus CQ-Mock controls (n = 8). The plasma levels of IFNy, TNF-α, IL2, IL6, IL4, IL5 and IL10 were measured 4 days after infection (post-challenge) with 10^5^ iRBC. *P < 0.05; **P < 0.01; ***P < 0.001 (Kruskal- Wallis test with Dunn’s Multiple Comparison).

Cytokine levels were assessed in sera of immunized animals before and on day 4 after the infectious challenge with iRBCs. Before challenge, levels of measured cytokines in the serum of control and immunized animals were low and no differences were observed between the individual groups analyzed (Table S1). On day 4 after iRBC challenge, the Th1 cytokine levels (IFNγ, TNFα and IL2) were significantly increased in CQ-RAS animals compared to the control CQ-Mock group (Fig. 6), with the most prominent increase in the levels of IFNγ. No significant difference was found between the CQ-CPS and CQ-Mock groups.

Regarding the Th2 cytokine IL4, an increase was found in CQ-RAS but not in CQ-CPS immunized mice (Fig. 6). IL5, another member of the Th2 cytokines, was significantly elevated in both immunization groups after challenge. For IL6 we observed comparable levels in CQ-RAS and CQ-Mock animals, however significant lower concentrations for the CQ-CPS group. Finally, an increase in IL10 was notable in both CQ-RAS and CQ-CPS immunized groups (Fig. 6).

## Discussion

Our investigations in the rodent model demonstrate that a single i.v. or s.c. whole-parasite immunization confers complete protection against ECM in the rodent model. Protection against ECM is mediated by stage-transcending, blood-stage directed immune responses both in the CPS and RAS immunization model.

This finding is of immediate clinical importance, as there are several ongoing clinical studies to assess the efficacy of whole-parasite immunization against human *Pf* malaria. The concept of whole-parasite immunization has been deployed to achieve complete, sterile immunity to malaria infection. Such high degree of protection is difficult to obtain and the requirement of intravenous injections of high SPZ numbers on multiple occasions^12,23,24^ remains a significant obstacle regarding translation towards routine vaccination. Recent reports suggest that sterile protection could be limited to experimental settings where whole-parasite immunization and infectious challenge is performed with the same homologous parasite strain. Challenge with a second, heterologous strain resulted in breakthrough infection in several trials^25–27^ and was also reported from a first assessment in the field^28^.

Prevention of severe malaria represents a less ambitious, yet possibly more feasible vaccination approach. Our findings from the rodent model suggest that a single whole-parasite immunization might be sufficient to induce protection against severe malaria disease.

For obvious ethical reasons, it is difficult if or even impossible to assess protective efficacy against severe malaria in humans. It was however reported that protection against severe malaria is acquired by only one or two natural infections^4^. It was also shown that CPS immunization leads to immune recognition of blood-stage parasites in humans. Bijker *et al*. previously subjected CPS-immunized and non-immunized volunteers to an asexual *Pf* blood-stage challenge. The asexual blood-stage challenge did induce significantly higher plasma concentrations of IFNγ in CPS-immunized individuals, suggesting that immune recognition of blood stage parasites is induced during human CPS immunization^13^. In addition, a study by Nahrendorf *et al*. confirmed the generation of memory B-cell and antibody responses against both pre-erythrocytic and cross-stage antigens in CPS immunization^29^. Thus, whole-parasite immunization induces blood-stage responses in humans. CPS immunization under CQ or DP chemoprophylaxis ensures exposure to both liver and early blood-stage parasites as both drugs exclusively target and eliminate *Plasmodia* parasites during blood stage development^10,14^. It is therefore not surprising that blood-stage directed immune responses have been reported both after rodent and human CPS immunization^13,30^. In RAS immunization, SPZ arrest during liver stage development and it is less clear if, and to what extent, immune recognition of blood-stage parasites is developed. Stage-transcending immune responses have previously been reported following the immunization with late liver-stage arresting genetically-attenuated parasites (GAP)^31,32^, which may be explained by a substantial overlap of antigen pools between late-liver-stage and blood-stage parasites^33^. In comparison to late liver-stage GAP, RAS arrest at various, generally earlier time points during intrahepatic development. Only immunization with late-liver-stage arresting GAP, but not RAS, conferred a high degree of protection against blood-stage challenge^31^. Unlike to CPS immunization, antibodies to asexual erythrocytic stages were undetectable in human volunteers after *Pf* RAS vaccination^24^.

In our study, protection against ECM and reduced blood-stage development was achieved both in the RAS and the CPS immunization model. Our data suggests that resistance to ECM might be obtained by differing immune mechanisms in RAS and CPS immunized animals. Blood-stage directed antibodies were only detected after CPS immunization, whereas cellular immune responses were observed in the blood of both CPS and RAS immunized mice. Th1 cytokine levels, in particular IFNγ, were more pronounced in RAS immunized mice. As inhibition of liver-stage development was only seen after a single RAS but not after CPS immunization, it can be assumed that the rate of liver-resident interferon-gamma-producing CD8^+^ T cells might be higher after RAS immunization, whereas blood-stage specific humoral immune responses are more pronounced in CPS immunized mice. Despite those immunological differences, a single immunization resulted in a comparable delay of blood-stage parasite development in the CPS and RAS model. The combination of pre-erythrocytic and blood-stage directed immunity might represent an important and unique benefit of whole-parasite vaccination.

The pathological findings in ECM in many ways resemble the findings in pediatric human cerebral malaria^19,34–36^. Therefore, the rodent malaria model, which predicted the finding of sterile protection in whole-parasite immunization, could represent a reliable model in regard to protection against severe disease and in particular cerebral malaria^37^.

We conclude that both single-dose RAS and CPS immunization has the potential to preclude the devastating consequences of cerebral malaria. Whole-parasite immunization, administered in few or even a single dose, could represent a simple and feasible method to protect humans living in endemic areas against fatal *P*. *falciparum* infection.

## Materials and Methods

### Ethics Statement

All animal experiments were performed according to FELASA category B and GV-SOLAS standard guidelines. Animal experiments were approved by German authorities (Regierungspräsidium Karlsruhe, Germany, § 8 Abs. 1 Tierschutzgesetz (TierSchG)).

### Animals, drug administration, radiation of SPZ and immunization procedure

All experiments were carried out with the rodent parasite *Plasmodium berghei* ANKA (*Pb* ANKA) in inbred C57BL/6 J mice. Female C57BL/6 J mice aged between 6–8 weeks were obtained from Janvier Labs, France. *Pb* ANKA SPZ were isolated by dissection of salivary glands from female *Anopheles stephensi* mosquitoes at day 19–25 after bloodmeal infection.

For immunization under chemoprophylaxis (CPS: Chemoprophylaxis with SPZ), experimental mice were either immunized by intravenous (i.v.) injections of 3 × 10^4^ SPZ in a total volume of 100 µl PBS at the beginning of continuous chloroquine (CQ) drug supply in drinking water (CQ-CPS i.v.), or by subcutaneous (s.c.) injection of 10^5^ SPZ in 100 µl PBS supplemented with 100 µg histamine concomitant with intraperitoneal piperaquine (PPQ) administration (PPQ-CPS sc), respectively.

For immunization with radiation-attenuated SPZ (RAS), SPZ were treated by exposure to 150 Gy of γ-radiation (^137^Cesium source, University Hospital Heidelberg, Germany). Experimental animals were immunized by i.v. administration of 3 × 10^4^ RAS. The timeline of different experimental immunizations is illustrated in Fig. S1.

### Clinical assessment by Rapid Murine Coma and Behavioral Scale

For clinical evaluation of neurological symptoms, malaria-infected mice were assessed daily starting from day 6 until day 14 after infection for ten parameters of cerebral symptoms according to the previously described Rapid Murine Coma and Behavioral Scale (RMCBS)^17^. ECM was reported in mice that died or showed symptoms of severe neurological disease, defined as a RMCBS <5.

### Assessment of parasitemia levels

Starting from day 2 (infection challenge with iRBC) or day 3 (infection challenge with SPZ), daily thin blood smears were obtained from all mice and labeled with running numbers to mask the treatment group to the slide-reading investigators. Blood smears were stained in Giemsa. Parasitemia levels were assessed by counting the number of visible parasites in 10 microscopic light fields. In slides with low number of parasites (<2 per microscopic light field), we increased the number of assessed light fields to 20. In the experiment presented in Supplementary Figure 3, parasitemia was assessed by two independent investigators.

### Evans blue extravasation

We injected mice intravenously (i.v.) with 0.15 ml of 2% Evans blue (Sigma) as soon as the RMCBS <5 was indicating severe neurological impairment (ECM positive mice), or at day 14 post infection (ECM negative mice). Mice were sacrificed 1 h after injection, perfused intracardially with 10 ml PBS and brains were weighed and placed in 2 ml of formamide (Merck) at 37 °C for 48 h to extract Evans blue dye from the tissue. Absorbance was measured at λ = 620 nm (Bio Rad SmartSpec 3000) as previously described^38^.

### *Ex vivo* MRI assessment

After transcardial perfusion with PBS brains were explanted and placed in a 15 ml Falcon tube (Corning) for MR imaging. MRI was performed on a 9.4 T small animal scanner (BioSpec 94/20 USR, Bruker, Ettlingen, Germany) using a volume resonator for transmission and a 4-channel-phased-array surface receiver coil. Microhemorrhages were determined by using a T2*-weighted flow compensated gradient echo sequence (TR/TE = 50/18 ms, FA = 12°, 80 µm isotropic resolution).

### *In vivo* imaging

The transgenic *P*. *berghei* line 676m1cl1 (*Pb* GFP-Luc_con_)^39^ was used for real-time *in vivo* imaging of liver-stage development. *Pb* GFP-Luc_con_ SPZ were injected into the tail vein of C57BL/6 J mice. Bioluminescence measurement was performed as previously described^40^. Luciferase activity was visualized immediately after the administration of D-Luciferin through whole-body imaging using an *in vivo* Imaging System (IVIS 100; Caliper Life Sciences, USA), Bioluminescence was acquired with an exposure time of 180 seconds and analyzed using Living Image 2.50.1 (Xenogen Corp., Hopkinton, MA, USA).

### *Ex vivo* imaging of brain parasite load

For *ex vivo* imaging of brains, C57BL/6 J mice were immunized with one-shot of 3 × 104 RAS (n = 12) or salivary gland debris (Mock; n = 8) and challenged 6 weeks later with 105 iRBC of Pb GFP-Luc_con_ WT parasites. Groups of mice were sacrificed and perfused intracardially with PBS at the day when Mock-injected mice showed ECM (day 7 after challenge; n = 7 for Mock-injected mice and n = 5 for one-shot RAS-vaccinated mice). For RAS-vaccinated animals, additional groups of mice were sacrificed at day 8 (n = 4) and day 9 (n = 3) post challenge. Brains were isolated and incubated for 10 minutes in a falcon tube containing 4 ml of PBS with 200 µl luciferin ((30 mg/ml stock solution); Synchem Laborgemeinschaft OHG, Germany), transferred in a petri dish and imaged. Bioluminescence was acquired after 1 minute exposure time with medium binning factor and FOV of 12.5 cm. All images were analysed using the ROI tool of the Living Image software (V2.50.1, Xenogen, Hopkinton, MA). The ROI is expressed as total flux (photons (p)/second (s)).

### ELISA

ELISA plates (MaxiSorb, Nunc) were coated with 5 µg/ml *P*. *berghei* late blood-stage (BS) lysate in bicarbonate/sodium carbonate coating buffer overnight at 4 °C. The lysate was generated from density-gradient enriched late blood stages treated with M-PER Mammalian Protein Extraction Reagent (ThermoFisher Scientific) for 3 h on ice followed by centrifugation at 15,000 g for 15 minutes at 4 °C. After coating overnight, ELISA plates were washed (PBS/0.05% Tween20) and blocked (6% BSA/PBS/0.05% Tween20) for 2 h at room temperature. Subsequently, respective sera were added in a 1:20 dilution in blocking buffer and incubated for 2 h at room temperature. After washing, the secondary antibody (anti-mouse IgG conjugated with HRP, Sigma-Aldrich) was added in a 1:5000 dilution for 1 h at room temperature. Before adding the substrate (SigmaFast OPD, Sigma-Aldrich), the ELISA plates were washed again. Finally, the absorbance at 450 nm was measured after 5–10 minutes of substrate incubation.

### Cytokine Bead Array Analysis

Cytokine Bead Array (CBA) analysis was performed using the LEGENDplex^TM^ Multi Analyte Flow Assay Kit (BioLegend) according to the manufacturers’ instructions. For determining the cytokine concentrations in serum, 50–100 µl of whole blood was obtained 4 weeks after the immunization procedure (2 weeks before challenge with iRBCs, pre-challenge), and on day 4 after challenge with iRBCs (day 4 post-challenge) from the eye background. Plasma was obtained by centrifugation and stored at −80 °C until usage. For the analysis, 2-fold diluted sera samples and serial dilutions of the respective cytokine standards were incubated with Capture Beads and Detection Antibodies for 2 h at room temperature in the dark on a plate shaker at 600 rpm. Subsequently, Streptavidin-phycoerythrin (SA-PE) was added and samples were incubated for additional 30 min at room temperature (plate shaker at 600 rpm) followed by a washing step with Wash Buffer. Finally, the bead pellet was re-suspended in Wash Buffer for measurement. 300 events/cytokine were acquired on a FACS Calibur. Cytokine concentrations were analyzed using the LEGENDplex^TM^ Data Analysis Software Version 7.0 (Biolegend).

### Cell preparation, cell staining and flow cytometry

The phenotype of CD4^+^ and CD8^+^ T lymphocytes in the blood was determined in mice immunized either by CQ-CPS (n = 8) or CQ-RAS regimen as described above and compared to non-immunized CQ-Mock mice (n = 8).

Two weeks before and four days after challenge with 10^5^ iRBCs injected intravenously, 50–100 µl blood was obtained from each mouse retroorbitally. Blood samples were centrifuged for 10 min at 3000 rpm (4 °C). Cell pellets were subjected to erythrocyte lysis using red cell lysis buffer (0.037 g EDTA, 1 g KHCO3, 8.26 g NH_4_Cl in 1 l ddH_2_O, pH 7.4). Finally, cells were re-suspended in RPMI complete medium (RPMI supplemented with 10% FCS, 1 × MEM NEAA (Gibco), 1 mM sodium pyruvate (Gibco), 5 ml penicillin/streptomycin (Gibco), and 10 μl of heparin). For pre-challenge analysis, cells were incubated in the presence of *P*. *berghei* blood-stage lysates for 24 h at 37 °C/5% CO_2_. Brefeldin A (Sigma-Aldrich) was added during the last 4 h in a final concentration of 10 µg/ml. As control, cells were incubated in RPMI complete medium without stimulus. Blood-stage lysates were obtained after three cycles of freezing (liquid N_2_) and thawing of gradient-purified late asexual stages. Subsequently, surface staining was performed using anti-mouse CD4 (clone: RM4-5/PE-Cy7/eBioscience), anti-mouse CD8 (clone: 53-6.7/FITC/eBioscience), anti-mouse CD62L (clone: MEL-14/PE/ eBioscience) and anti-mouse CD44 (clone: IM7/PerCP-Cyanine5.5/eBioscience) for 20 min on ice. Cells were washed with PBS before fixation with 2% PFA/PBS for 15 min at room temperature followed by an intracellular staining with anti-mouse IFNγ (clone: XMG1.2/APC-Cy7/BD Biosciences) in permeabilization buffer (0.1% BSA, 0.3% Saponin in PBS) for 20 min on ice. Finally, cells were washed and re-suspended in PBS (subsequent data acquisition) or 1% PFA/PBS and incubated for 5 min at room temperature in the dark, washed once with PBS and stored at 4 °C until data acquisition. For post-challenge analysis, cells were stained *ex vivo* using the surface markers as described above. Cells were measured using a FACS Canto I flow cytometer. All data were processed and analyzed using CellQuest Pro Software (version 6.0).

### Data assessment and statistical analysis

Data assessment and statistical analysis was performed using Prism Version 5.0b for Mac OS X (GraphPad Software Inc, La Jolla, CA, USA).

### Data availability

The data that support the findings of this study are available from the corresponding author upon reasonable request.

## Electronic supplementary material

Supplementary Dataset 1
