# Supplementary material for: Protection from experimental cerebral malaria with a single intravenous or subcutaneous whole-parasite immunization
Source: Sci Rep. 2018 Feb 15;8:3085. doi: 10.1038/s41598-018-21551-2 (PMC5814423; doi:10.1038/s41598-018-21551-2)
Supplement: Supplementary file 1 — Supplementary Dataset 1 [file 41598_2018_21551_MOESM1_ESM.doc]

# SUPPLEMENTARY MATERIALS

# Protection from experimental cerebral malaria with a single intravenous or subcutaneous whole-parasite immunization

Kirsten Heiss^§^ ^1,6^, Marion Irmgard Maier^§^ ^1,3^, Angelika Hoffmann ^4,5^, Roland Frank^1^, Martin Bendszus ^4^, Ann-Kristin Mueller ^1,3^ and Johannes Pfeil ^1,2,3^

^1^Centre for Infectious Diseases, Parasitology Unit, Heidelberg University Hospital, Germany

^2^Centre for Childhood and Adolescent Medicine, General Pediatrics, Heidelberg University Hospital, Germany

^3^German Centre for Infection Research (DZIF), Heidelberg, Germany

^4^ Department of Neuroradiology, Heidelberg University Hospital, Germany

^5^Division of Experimental Radiology, Department of Neuroradiology, Heidelberg University Hospital, Germany

^6^MalVa GmbH, Heidelberg, Germany

§ these authors contributed equally to this work

*To whom correspondence should be addressed: Johannes Pfeil, Im Neuenheimer Feld 430, 69120 Heidelberg, Germany, Tel. +49-6221-5637121, Email: Johannes.Pfeil@med.uni-heidelberg.de

**Table of Contents**

**I. Supplementary figures**

**Fig. S1** Immunization schedules and legend

**Fig. S2** ECM-free survival in CQ-RAS (n=8) or CQ-CPS (n=8) immunized mice and CQ-Mock controls (n=8).

**Fig. S3** Parasitemia levels in CQ-RAS (n=8) and CQ-CPS (n=8) immunized mice versus CQ-Mock controls (n=8).

**II. Supplementary tables**

**Table S1.** Cytokine concentrations (pg/ml) measured in the serum of CQ-Mock, CQ-RAS and CQ-CPS immunized mice before iRBC challenge. Shown are mean values ± SD.

**I. Supplementary figures**

**Fig. S1. Immunization schedules and legend**

I _CQ-CPS_: 3x10^4^ *Pb* ANKA SPZ i.v., 1mg Chloroquine (CQ) (saturation dose) administered intraperitoneally (i.p.), continuous administration of CQ-drinking water (CQ-DW) for 2 weeks.
I _DP-CPS_: 10^5^ *Pb* ANKA SPZ and 100µg histamine sc, single-dose of 0.25/2mg Dihydroartemisinin-Piperaquine (DP) ip.
I _RAS_: 3x10^4^ radiation-attenuated *Pb* ANKA SPZ i.v., 1mg Chloroquine (CQ) i.p. (saturation dose), continuous administration of CQ-DW for 2 weeks.
I_CQ- RAS_: 3x10^4^ radiation-attenuated *Pb* ANKA SPZ iv, 1mg Chloroquine (CQ) i.p. (saturation dose), continuous administration of CQ-DW for 2 weeks.
D _CQ_: 1mg CQ ip (saturation dose), CQ-DW for 2 weeks.
D _DP_: Single-dose of 0.25/2mg DP i.p.
C _SPZ_: Challenge with 10^3^ *Pb* ANKA SPZ i.v.
C _iRBC_: Challenge with 10^5^ *Pb* ANKA infected Red Blood Cells i.v.
B: Blood sampling for immunological analysis. Blood samples were obtained from CQ-CPS, CQ-RAS and CQ-Mock (“CQ-treated”) mice before and on day 4 after C _iRBC_.

**Fig. S2. ECM-free survival in CQ-RAS (n=8) or CQ-CPS (n=8) immunized mice and CQ-Mock controls (n=8).** On day 12 after challenge with 10^5^ iRBC, 7 out of 8 CQ-RAS and 7 out of 8 CQ-CPS immunized mice, but only 2 out of 8 CQ-Mock survived without developing ECM (Fishers exact-test: P = 0.01 for both CQ-RAS or CQ-SPZ versus CQ-Mock).

**Fig. S3 Parasitemia levels in CQ-RAS (n=8) and CQ-CPS (n=8) immunized mice versus CQ-Mock controls (n=8).** On days 4 and 5 after infection with 10^5^ iRBC, CQ-RAS and CQ-CPS immunized mice were found with lower parasitemia levels in comparison to the CQ-Mock control mice (* P < 0.05 ** P < 0.01 *** P < 0.001, CQ-RAS or CQ-CPS versus CQ-Mock, Mann-Whitney-U-test). We could not observe any significant difference in parasitemia between CQ-RAS or CQ-CPS immunized mice.

**II. Supplementary Tables**

**Table S1. Cytokine concentrations (pg/ml) measured in the serum of CQ-Mock, CQ-RAS and CQ-CPS immunized mice before iRBC challenge. Shown are mean values ± SD.**

|  | **CQ-Mock** | **CQ-RAS** | **CQ-CPS** |
| --- | --- | --- | --- |
| IFNγ | 9,8 pg/ml ± 1,6 | 8,7 pg/ml ± 2,7 | 10,1 pg/ml ± 1,8 |
| TNFα | 12,3 pg/ml ± 2,0 | 11,3 pg/ml ± 3,6 | 10,0 pg/ml ± 4,2 |
| IL2 | 4,0 pg/ml ±1,0 | 3,1 pg/ml ± 1,5 | 3,1 pg/ml ± 1,1 |
| IL4 | 3,7 pg/ml ± 2,3 | 2,5 pg/ml ± 1,1 | 3,8 pg/ml ± 0,8 |
| IL5 | 8,0 pg/ml ± 2,5 | 5,4 pg/ml ± 1,4 | 5,6 pg/ml ± 0,9 |
| IL6 | 3,3 pg/ml ± 1,7 | 2,4 pg/ml ± 0,4 | 2,6 pg/ml ± 0,5 |
| IL10 | 21,6 pg/ml ± 5,7 | 19,4 pg/ml ± 10,3 | 16,4 pg/ml ± 4,2 |
